# Combined effects of salicylic acid and furosemide and noise on hearing

**DOI:** 10.1186/1745-6673-7-1

**Published:** 2012-01-22

**Authors:** Marrigje A de Jong, Cahtia Adelman, Melissa Rubin, Haim Sohmer

**Affiliations:** 1 Dept. of Medical Neurobiology (Physiology); Institute for Medical Research - Israel-Canada, Hebrew University-Hadassah Medical School, POB # 12272 Jerusalem 91120 Israel; 2 Speech & Hearing Center, Hadassah University Hospital, POB # 12000 Jerusalem 91120 Israel; 3 Touro College; 27-33 W 23rd St, New York, NY. 10010, USA

**Keywords:** noise induced hearing loss, salicylic acid, furosemide, cochlear amplifier, ABR, free radicals, prestin, endocochlear potential

## Abstract

**Background:**

A major cause of the hearing loss following exposure to intense noise involves release of free radicals resulting from the elevated metabolism. The free radicals induce damage to several of the components of the cochlear amplifier including the outer hair cells and indirectly to the transduction currents. Salicylic acid induces a reversible hearing loss since it binds to the motor protein prestin in the outer hair cells, reducing electromotility. Furosemide also induces a reversible hearing loss since it reduces the endocochlear potential which is a major component of the cochlear transduction currents. On the other hand, each of these drugs also provides protection from a noise induced hearing loss if they are injected just before a noise exposure, probably as a result of the decreased metabolism induced in their presence, with release of lower levels of free radicals. In this study, both drugs were administered in order to assess whether their protective effects would be additive.

**Methods:**

The study was conducted on normal hearing albino mice of the Sabra strain. They were injected with either salicylic acid alone (N = 11), or furosemide alone (N = 14), or both together (N = 14), or with saline control (N = 11) and exposed to broad band noise for 3.5 hours. An additional group of 9 mice was injected with both salicylic acid and furosemide, but not exposed to noise. The degree of the resulting hearing loss was assessed by recording thresholds of the auditory nerve brainstem evoked responses to broad band clicks before the injections and noise, and 7, 14 and 21 days after.

**Results:**

The noise induced hearing loss in the mice injected with salicylic acid alone or furosemide alone was smaller than in those injected with saline, i.e. these drugs provided protection, as in previous studies in this laboratory. There was no threshold elevation after two weeks in the mice injected with both drugs without noise exposure, i.e. the effects of the two drugs given together was reversible. On the other hand, there was a significant hearing loss (i.e. threshold elevation) in the group which received both drugs and was also exposed to noise, with mean threshold elevations of 38.8 ± 19.0 dB and 28.3 ± 11.7 dB 7 days after noise exposure.

**Conclusions:**

This result is very surprising, if not paradoxical. Drugs which provide protection from a noise induced hearing loss when administered alone, not only do not provide protection when given together, but also induce a greater hearing loss when accompanied by noise. This observation may be related to the finding that the depression of the endocochlear potential normally caused by furosemide is reduced in the presence of salicylic acid, so that the protection usually provided by furosemide is not present when it is administered together with salicylic acid. Thus it seems that each drug may interfere with the protective action of the other when coupled with noise.

## Background

Hearing loss is a debilitating condition which leads to severe reductions in social interactions. This loss can be caused by external factors such as noise exposure and several drugs such as antibiotics and loop diuretics, and on intrinsic factors such as presbyacusis. Therefore attempts have been made to determine the mechanisms of the hearing loss caused by each of these agents as a prerequisite to the design of ways to prevent or reduce the loss. For example, it has been suggested that exposure to intense noise causes a hearing loss, which can be temporary or permanent (depending on the intensity and duration of the exposure), due to the release of reactive free radicals which cause a lesion to one or more components of the cochlear amplifier, with outer hair cell (OHC) loss and damage to their stereocilia, as well as decreased transduction currents [1,2]. The drug salicylic acid (the active ingredient in aspirin) causes a temporary hearing loss by binding to the motor protein prestin, and thereby reduces the electromotility of the OHC, which is also a component of the cochlear amplifier [3]. Furthermore, the loop diuretic furosemide also induces dysfunction of the stria vascularis, causing a reduction in the endocochlear potential (a major driving force for the transduction currents and for the cochlear amplifier), and a reversible hearing loss [4].

On the other hand, it has been shown that the administration of either of these drugs, salicylic acid [5] and furosemide [6], just before a noise exposure leads to a significant reduction in noise induced hearing loss (NIHL), providing protection from that noise exposure. That is, the resulting noise induced loss in animals given either of these drugs at appropriate times before the exposure is significantly smaller than that in the animals exposed to the same noise without prior drug administration [5,6]. It has been suggested that the mechanism of this protection involves the temporary reversible depression of the cochlear amplifier induced by each of these drugs, so that metabolism is reduced and lower levels of free radicals are released during the noise exposure.

The present study was designed to analyze these apparently contradictory effects: drugs which themselves induce a hearing loss, can nevertheless protect against a noise induced HL. The study involved subjecting animals simultaneously to the three agents (salicylic acid, furosemide and noise) and then determining the degree of any resulting hearing loss. Would the protective effects of the drugs be additive, as has been shown when salicylic acid is administered together with N-acetylcysteine [7], or would the resulting hearing loss be no greater than that following each drug alone? In the first part of the study, the drug doses were identical to those in previous studies in this laboratory. The surprising results from this part led to repeating it in a second set of mice with lower doses.

## Materials and methods

### Animals

The study was conducted on albino male mice of the normal Sabra strain, obtained from Harlan Laboratory, Israel (n = 59 divided into two sets) at an initial age of 6-7 weeks and a mean body weight of 39.6 ± 3.5) grams. They all had normal hearing, defined as auditory nerve brainstem evoked response (ABR) thresholds to broadband clicks delivered by an insert earphone, of 65 dB peak equivalent (pe) SPL or better.

### Anesthesia

All mice were anesthetized with 4.5 mg/kg Avertine intraperitoneally (IP) prior to ABR recordings and additional anesthesia was given when required in order to maintain areflexia.

### Auditory Brainstem Response

ABR was recorded in each mouse in response to alternating polarity broadband clicks presented by an insert earphone in the left external ear canal (Navigator Pro System, Biological Systems Corporation, Mundelein, Illinois, USA). Broad band click stimuli were used in order to rapidly screen auditory function over a broad range of frequencies in a large number of animals, and to enable comparison with previous studies [5,6]. In addition, rapid screening was essential for the successful conduction of the study, as there were tight time restrictions involving drug injection followed by exposure of the animals to noise after fixed time periods. Recording subdermal needle electrodes were inserted in the skin at the vertex between the ears with reference to the chin and a ground electrode in the left hind limb.

Stimuli were presented at a rate of 20/s from a maximal intensity of 120 dB pe SPL to 5 dB below threshold, in steps of 5 dB. Threshold was defined as the lowest stimulus intensity with a clear repeatable component (usually the first wave) in at least 2 out of 3 ABR recordings. Initial ABR was assessed 2-3 days before noise exposure and repeated ABR tests were done 7, 14 and 21 days after noise exposure. The experimental protocol was evaluated and approved by the Hebrew University Hadassah Medical School Animal Care and Use Committee (MD-10-12660-3).

### Drugs

Both drugs were administered systemically by intraperitoneal (IP) injections. In the first part of the study (Set A, 31 mice), salicylic acid was administered at a dose of 350 mg/kg and furosemide at 100 mg/kg. In the second part (Set B, 28 mice), the dose of salicylic acid was reduced to 300 mg/kg, and furosemide to 50 mg/kg. The final doses for the second part of the study were determined based on a preliminary study in 17 additional mice receiving different regimens of salicylic acid and/or furosemide with and without noise exposure. Previous studies showed that a plateau of the depressant effect of salicylic acid by at least 30 dB was apparent from 60 minutes to 180 minutes after the injection [5] and that the depressant effect of furosemide (26 dB) was observed after 30 minutes,. increasing to a maximum mean ABR threshold elevation of 37 dB after 60 minutes and recovering 3 hours later (ie, 4 hours after the injection) [6]. Therefore, in the group receiving both salicylic acid and furosemide, these drugs were administered 90 and 30 minutes respectively before the noise exposure, so that the maximum period of depression of the cochlear amplifier induced by each drug would overlap with each other and with the noise exposure. In order to avoid possible protection from NIHL by the restraint and stress resulting from the handling and injection of the drugs IP [8], each animal in both parts of the study received the same total number of injections, supplementing them with saline when necessary. Therefore the animals to be injected with salicylic acid alone were given that drug 90 minutes before the noise, supplemented by saline 30 minutes before the noise. On the other hand, the animals to be injected with furosemide alone were given saline 90 minutes before the noise, followed by furosemide 30 minutes before the noise.

### Experimental groups in both parts of the study

Group I: Saline + noise. Eleven mice received two injections of saline, 90 and 30 minutes before they were exposed to noise.

Group II: Salicylic acid + noise. Eleven mice were injected with salicylic acid 90 minutes before the onset of noise exposure, at a dose of 350 mg/kg (n = 5, Set A) or 300 mg/kg (n = 6, Set B). This was followed by an injection of saline 30 minutes before the onset of noise exposure.

Group III: Furosemide + noise. Fourteen mice were given saline 90 minutes before the onset of noise exposure and then furosemide at a dose of 100 mg/kg (n = 8, Set A) or 50 mg/kg (n = 6, Set B), 30 minutes prior to the noise exposure.

Group IV: Salicylic acid + furosemide + noise. Fourteen mice were administered salicylic acid 350 mg/kg (n = 8, Set A) or 300 mg/kg (n = 6, Set B), 90 minutes before the onset of noise exposure, followed by furosemide 100 mg/kg (n = 8, Set A) or 50 mg/kg (n = 6, Set B), 30 minutes before the onset of noise exposure.

**Figure 1:**
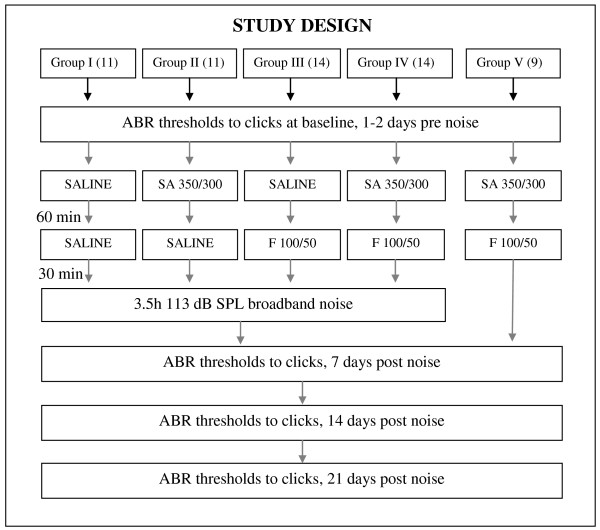
**Study design**. Flow chart for the different groups I - V (number of animals) with the order of auditory brainstem response (ABR) measurements, the time in minutes (min) between administration of saline, salicylic acid (SA: 350 mg/kg Set A or 300 mg/kg Set B) and furosemide (F: 100 mg/kg Set A or 50 mg/kg Set B) and noise exposure.

Group V: Salicylic acid + furosemide without noise. Three mice in the first group (Set A) received an injection of 350 mg/kg salicylic acid followed by an injection of 100 mg/kg furosemide after 60 minutes. In the second part of the study, six mice (Set B) received 300 mg/kg salicylic acid in combination with 50 mg/kg furosemide. They were not exposed to noise. The study design is illustrated in Figure 1.

### Noise exposure

The mice were exposed to broadband noise in both parts of the study at an intensity of 113 dB SPL for 3.5 hours while they were awake. The noise peaked at 2 kHz and was 14 dB down at 250 Hz and 15 dB down at 8 kHz [9] in both parts of the study at an intensity of 113 dB SPL for 3.5 hours while they were awake. This intensity and duration are similar to that in previous studies in our laboratory and produce an intermediate degree of permanent threshold shift (PTS) so that any protection by the drugs could be assessed (i.e. a shift that is not too small, indicating total protection; and not too large causing a "ceiling effect"). The intensity and spectrum of the noise were periodically evaluated with a Bruel & Kjaer precision integrating sound level meter (type 2218) with a third octave filter [9]. The animals, in 2 cages (11 animals in each cage) from the experimental and control groups, were exposed together to the noise generated by a loudspeaker positioned centrally about 30 cm above them. With onset of the noise, the animals appeared agitated, but after that initial phase, they settle down after a few minutes and resume their normal behavior, moving in the small cage and drinking and eating and resting alternately without huddling together, so that they were equally exposed to the noise. ABR thresholds were determined in each of the mice one week after the exposure in set A, and in set B also two and three weeks after the injections and noise exposure, and their significance was assessed with paired t-tests and one way ANOVA.

## Results

Seven days after the noise exposure, ABR thresholds were significantly (paired t-tests; P < 0.05) elevated in each of the groups exposed to noise (see table for mean ± SD thresholds and their shifts). The largest threshold elevation after one week was found in the group given all three agents (group IV-salicylic acid + furosemide + noise): 38.8 ± 19.0 dB in Set A (salicylic acid 350 mg/kg with furosemide 100 mg/kg) and 28.3 ± 11.70 dB in Set B (salicylic acid 300 mg/kg with furosemide 50 mg/kg). There was no significant difference in the threshold shifts between the two groups that were exposed to all three agents: two drugs and noise, i.e. groups IV, and the saline group (i.e. group I) exposed to noise (ANOVA). There was also no significant difference in threshold elevation between the two groups (sets) which received different doses of the two drugs. When comparing the threshold shifts in the mice that were exposed to noise after three weeks to those after two weeks, there was no change in the threshold shifts to noise, i.e. the threshold shifts had stabilized, and therefore this was considered a permanent threshold shift. The group receiving both drugs, but without noise exposure (group V), showed no significantly elevated thresholds, with almost complete recovery of the ABR thresholds after three weeks. The results are presented in table 1.

**Table 1:** ABR thresholds (± SD) in the different groups at baseline and 7 and 21 days later.

Experimental group	Set A (SA 350 mg/kg, F 100 mg/kg)				Set B (SA 300 mg/kg, F 50 mg/kg)					
	
	n	Threshold baseline (dB)	Threshold day 7 (dB)	Threshold shift	n	Threshold baseline (dB)	Threshold day 7 (dB)	Threshold shift	Threshold day 21 (dB)	Threshold shift
**I: Saline **+ **noise **exposure	7	52.1 ± 10.4	82.1 ± 17.5	34.3 ± 17.7	4	62.5 ± 2.9	90.0 ± 16.8	27.5 ± 18.9	91.3 ± 18.9	28.8 ± 19.3

**II: SA only + noise **exposure	5	48.0 ± 9.0	73.0 ± 18.2	25.0 ± 17.0	6	59.2 ± 5.8	78.3 ± 16.0	19.2 ± 13.9	76.7 ± 15.4	15.8 ± 10.2

**III: F only **+ **noise **exposure	8	55.0 ± 7.1	76.9 ± 17.1	21.9 ± 13.4	6	59.2 ± 5.8	80.0 ± 8.4	20.8 ± 7.4	81.7 ± 11.3	22.5 ± 16.0

**IV: SA + F + noise **exposure	8	51.9 ± 12.2	88.8 ± 14.8	38.8 * ± 19.0	6	59.2 ± 5.8	87.5 ± 15.7	28.3 * ± 11.7	90.0 ± 16.1	30.0 * ± 13.4

**V: SA + F **no noise exposure	3	50.0 ± 8.7	58.3 ± 5.8	6.7 ± 5.8	6	60.8 ± 3.8	68.3 ± 15.4	10.0 ± 12.3	63.3 ± 5.8	2.5 ± 4.2

Different drug doses were administered to the two sets (A and B) of mice.

* There is no significant difference in threshold shift between the mice receiving all 3 agents (group IV; SA + Furosemide + noise) and those receiving saline before noise (group I). (P > 0.6)

SA: salicylic acid, F: furosemide, dB: decibel, n: number of mice

## Discussion

In the present study, we were able to confirm our earlier findings with the same species, using the same noise regimen, that a single injection of salicylic acid 350 mg/kg [5] and a single injection of furosemide 100 mg/kg [6], each by itself, administered just before a noise exposure, led to a reduction in the permanent noise induced threshold shift assessed with ABR, probably as a result of the depression of the cochlear amplifier during the noise exposure. We chose to assess the effects of these agents (drugs and noise) by recording ABR thresholds and not with distortion product oto-acoustic emissions (DPOAE) as a result of our observation that in this strain of mice, following only 30 minutes of exposure to the noise applied in the present study, the DPOAE was totally absent. Furthermore, it has been shown [5] that the significant protective effect of salicylic acid was only apparent when administered before, but not after, the noise exposure. On the other hand, if the protective effect of salicylic acid were due to its anti-oxidant properties, then it would have been effective when injected after the noise. The magnitude of this protection was overall similar to that in the earlier studies with salicylic acid and with furosemide [5,6]. Since the durations of the depressant effects of each of the drugs more or less overlapped with the duration of the noise exposure (3.5 hours), it is likely that the mechanism of protection by each drug alone was a result of the depression of the cochlear amplifier. In addition, the ABR threshold shift seen in the present study following the combined administration of salicylic acid and furosemide without noise was reversible after two weeks. Therefore, the finding in both parts of the study that the combined administration of both drugs at the appropriate times before the noise exposure caused a large permanent threshold elevation was very surprising and paradoxical. We had expected either that in combination the drugs would not lead to a greater protection than that accompanying each of them alone, or that there would be augmented protection, i.e. an additive effect. As a matter of fact, in this present study the opposite seems to be true. The protective effects of salicylic acid and furosemide were completely absent when the drugs were administered simultaneously before the noise exposure. An attempt must be made to understand these unexpected results.

Several studies have demonstrated interactions between drugs, influencing their ototoxicity. For example synergy between salicylic acid and N-acetylcysteine has been demonstrated; a greater degree of protection from noise is seen when administered together, than with each drug alone [7]. It has also been shown that furosemide augments the ototoxicity of aminoglycoside antibiotics e.g. kanamycin [10]. On the other hand, in animals injected with salicylic acid before administration of furosemide, a smaller reduction in the endocochlear potential was found compared to those injected with furosemide alone [11], resulting in a smaller threshold elevation in the animals given both drugs. Other organic acids, such as penicillin and probenecid [12] also have been shown to reduce the magnitude of change in the endocochlear potential resulting from furosemide injection.

It is not likely that the combined drug injection disrupted the timing of the protective effects of each drug, since we have found similar threshold elevations in other experiments in which mice were given salicylic acid and furosemide [13].

In addition, each drug by itself and in combination, but without noise exposure, still leads to a reversible hearing loss, with recovery after two weeks.

## Conclusions

In final analysis, the administration of the two drugs together before exposure to noise (each providing protection from NIHL by itself) may lead to their mutual interference, so that there is less protection from a noise exposure. Future studies should investigate the mechanism of this surprising result.

## List of abbreviations

ABR: auditory nerve brainstem evoked response; SA: salicylic acid; F: furosemide; NIHL: noise induced hearing loss; dB: decibel; PTS: permanent threshold shift; OHC: outer hair cell.

## Competing interests

The authors declare that they have no competing interests.

## Authors' contributions

MAdJ conducted the experiments and contributed to writing the manuscript. CA contributed to the conduction, planning and writing. MR helped in the experiments. HS conceived the design, planning and composed the draft. All authors have read and approved the final manuscript.
